# Supplementary figures and images for: Assessing the potential of plains zebra to maintain African horse sickness in the Western Cape Province, South Africa
Source: PLoS One. 2019 Oct 31;14(10):e0222366. doi: 10.1371/journal.pone.0222366 (PMC6822716; doi:10.1371/journal.pone.0222366)

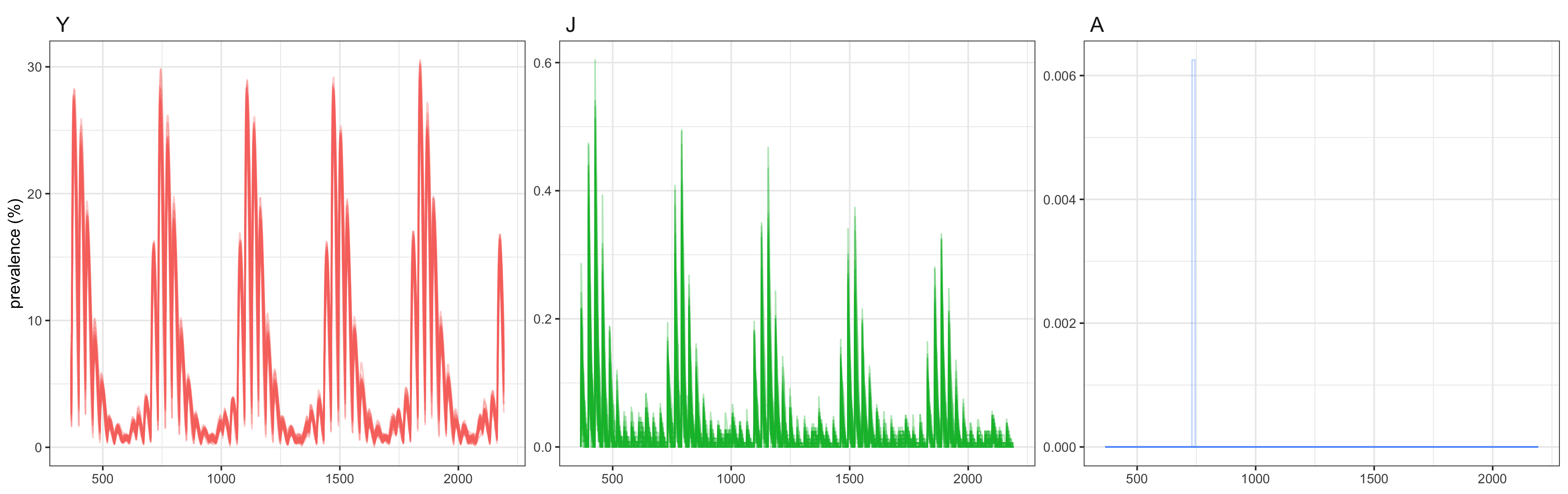

Supplement: S1 Fig — Temporal variation in the proportion of foals (Y), juveniles (J), and adults (A) that are infectious following 40 independent incursion events occurring on January 1st 2012. Here is shown the prevalence one year after the initial introduction event, assumed representative to the endemic circulation of AHS in the KNP population of plain zebra. Note the difference of scales in the y-axis. (TIFF) [file pone.0222366.s001.tiff]

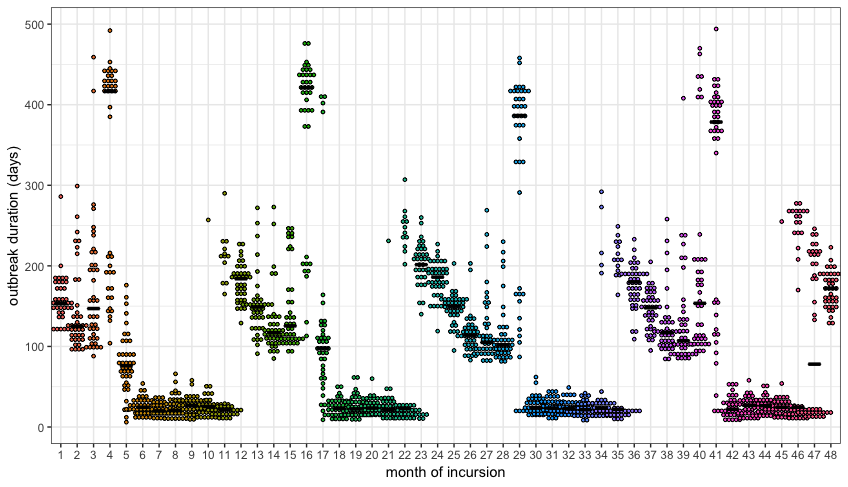

Supplement: S2 Fig — Distribution of the duration of the outbreaks generated from 40 independent incursion events occurring at the first day of each month between January 2012 and December 2015. Here we considered incursions occurred in the largest population of plains zebra reported in the WCP (N = 168) at the start of each simulation. Horizontal black bars show the mean outbreak duration. (TIFF) [file pone.0222366.s002.tiff]

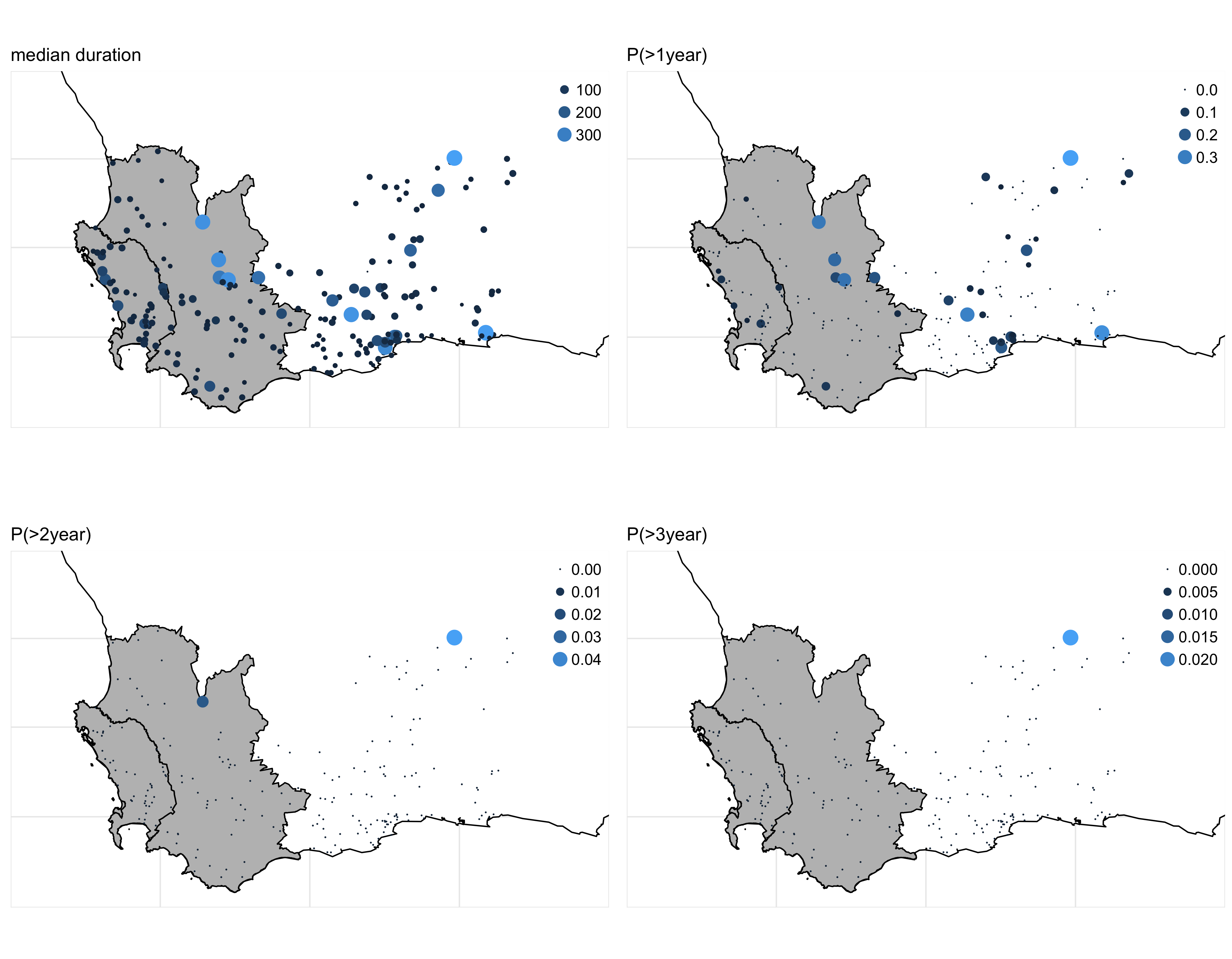

Supplement: S3 Fig — Risk of persistence was computed using either the median outbreak duration and the probabilities that AHS outbreaks would persist for >1 year, >2 years and >3 years. Risk measures were computed over 40 independent AHS introductions in all individual holdings. Here AHS was consistently introduced in April 2012. (TIFF) [file pone.0222366.s003.tiff]

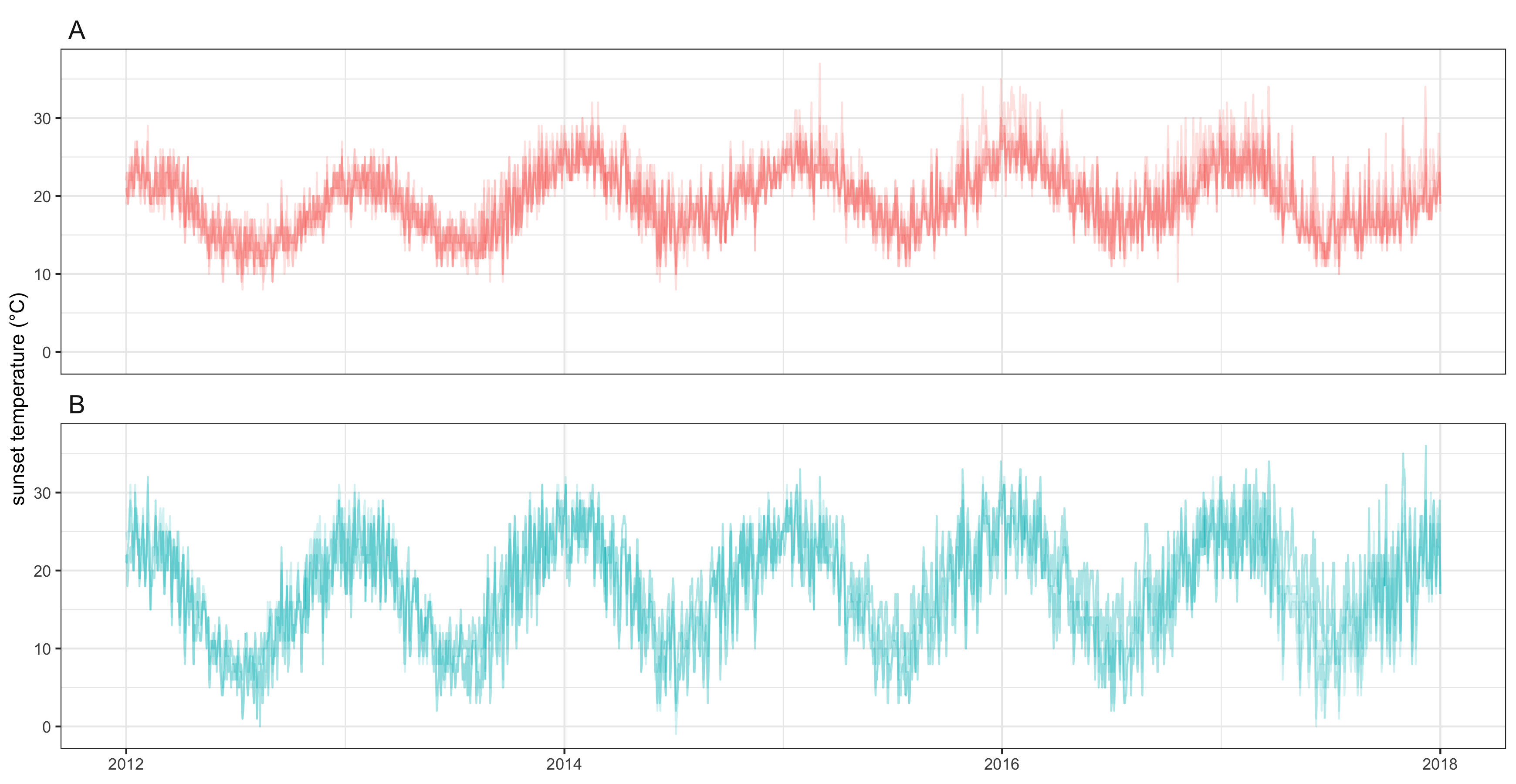

Supplement: S4 Fig — Daily variations in sunset temperatures for all sites considered in this work and driving holdings in (A) group A and (B) group B as defined in Fig 6. (TIFF) [file pone.0222366.s004.tiff]

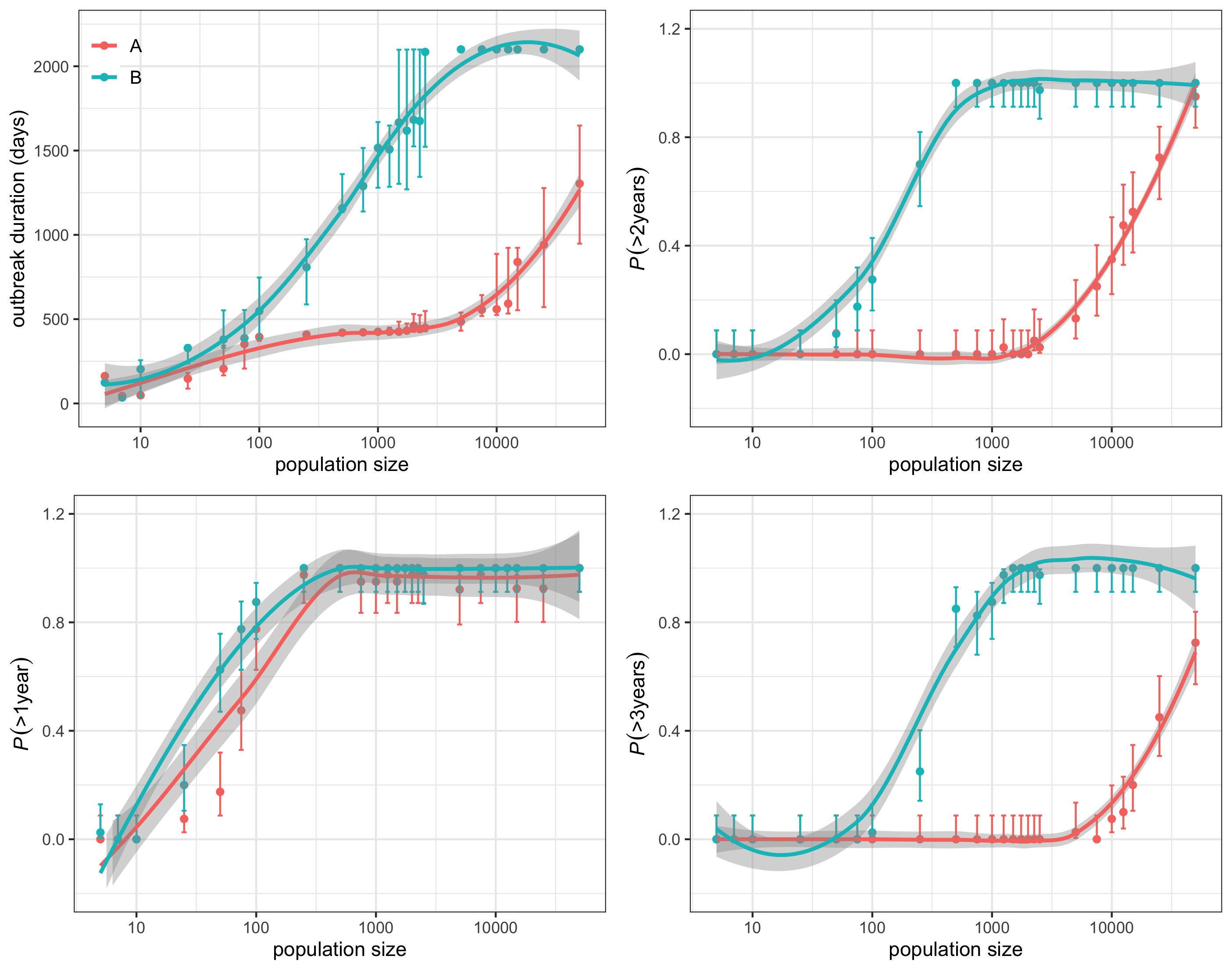

Supplement: S5 Fig — Risk of persistence was computed using either the median outbreak duration and the probabilities that AHS outbreaks would persist for >1 year, >2 years and >3 years. Risk measures were computed over 40 independent AHS introductions in considered scenarios. AHS was consistently introduced in April 2012 and considering an initial population density consistent to the median density reported in the WCP. Here is shown the outcomes for incursions in a typical holding located in either climatic zone A or climatic zone B. (TIFF) [file pone.0222366.s005.tiff]

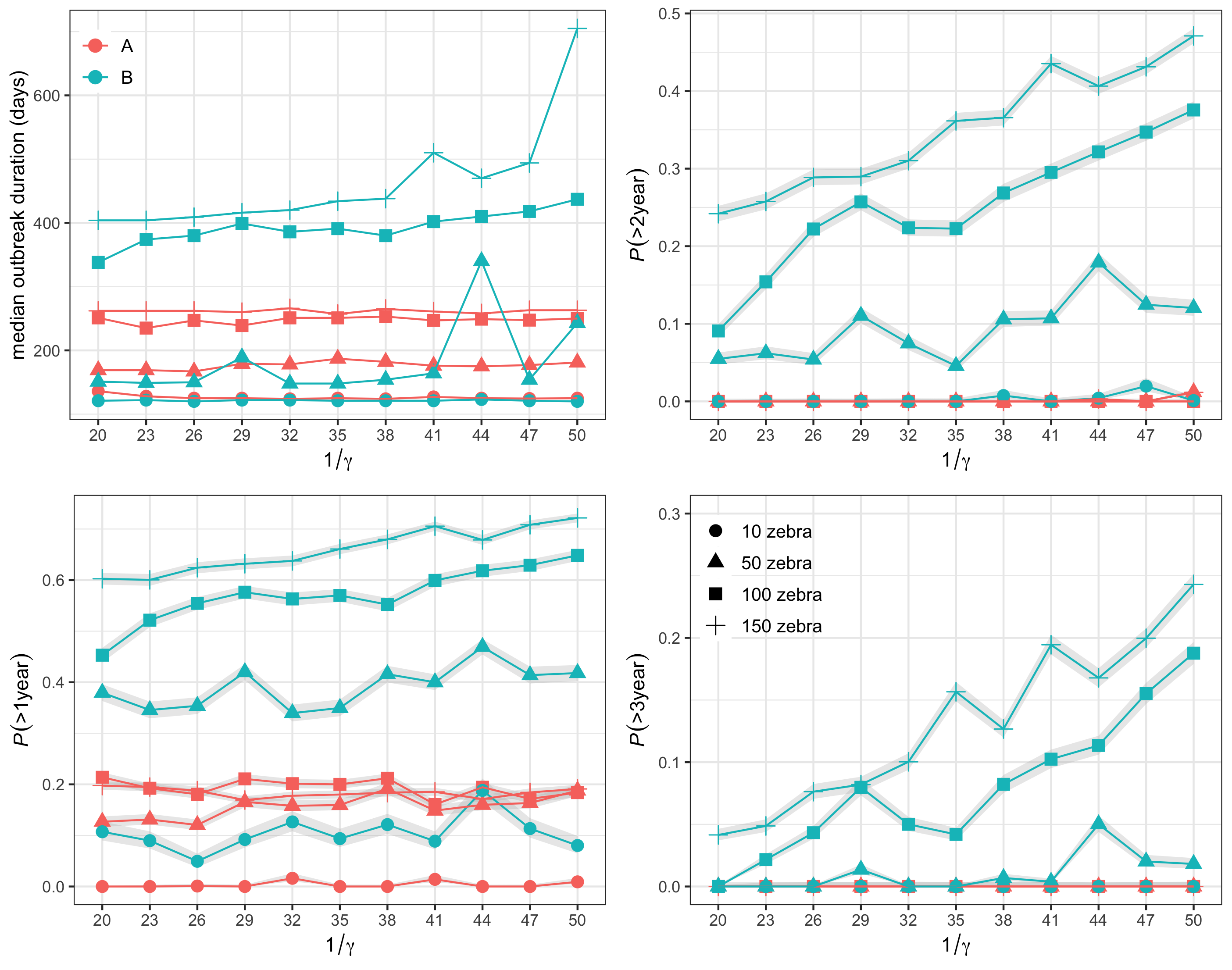

Supplement: S6 Fig — Risk of persistence was computed using either the medium outbreak duration and the probabilities that AHS outbreaks would persist for >1 year, >2 years and >3 years. Risk measures were computed over 40 independent AHS introductions in considered scenarios. AHS was consistently introduced in April 2012 and considering an initial population density consistent to the median density reported in the WCP. Here is shown the outcomes for incursions in a typical holding located in either climatic zone A or climatic zone B. (TIFF) [file pone.0222366.s006.tiff]
